# Using Digital Data to Protect and Promote the Most Vulnerable in the Fight Against COVID-19

**DOI:** 10.3389/fpubh.2020.00296

**Published:** 2020-06-12

**Authors:** Rumi Chunara, Stephanie H. Cook

**Affiliations:** ^1^Department of Biostatistics, New York University School of Global Public Health, New York, NY, United States; ^2^Department of Computer Science & Engineering, New York University Tandon School of Engineering, New York, NY, United States

**Keywords:** COVID 19, disparities, digital, data, public health

## Introduction

The COVID-19 pandemic has illuminated the numerous, important ways in which digital data is critical in public health preparedness and response. Indeed, we are seeing a rapid increase in the types of data being generated and used to directly inform and evaluate public health policy, such as forecasting trends in disease incidence via changes in fever reports from aggregate digital thermometer data (1), assessing mobility decreases via aggregate mobile phone GPS data by city for evaluation of concordance with physical distancing interventions (2), and sourcing individual-level mental and behavioral health changes in relation to epidemic trends via smartphone surveys (3). Using data from such tools in infectious disease research is not new; satellite imagery have been used for ascertaining sub-city factors related to dengue incidence (4), mobile phone calling records for quantifying the impact of mobility in relation to malaria spread (5), and smartphone surveys to understand care-seeking, and other behaviors during influenza epidemics (6). However, this is a critical inflection point in the use of digital data for infectious disease research and practice; we are all witnessing how such data is actively being used to inform policies in real time, globally. Simultaneously, the latest COVID-19 research shows that there is elevated risk for older people, that per capita disease burden and relative healthcare system demand may be highest away from major population centers, and that there are disparate rates of testing and morbidity by racial group (7, 8). Given the advent and welcome sharing of digital data, we must do so in a manner that ensure the most pressing public health issues are addressed to mitigate further impact of the pandemic. Indeed, attention to what types of data are used and who is represented in the data will significantly advance current practice by improving inferences to better avert illness and death in the population.

The digital divide shows that the most vulnerable (e.g., elderly, certain socioeconomic or cultural groups) may not often have access to the same digital tools, nor follow the same patterns of illness and reporting as other populations (9). This may present issues when population-level inferences are to be made from data that is generated from digital tools that are primarily used and generation by urban, younger and higher socioeconomic groups. In the current COVID-19 pandemic situation, assessments of the efficacy of social distancing interventions, or measurements of community disease burden would guide policy incorrectly if certain populations were omitted from such analyses. These issues are not novel, they reflect challenges highlighted in recent work stressing that when statistical, machine learning, and artificial intelligence methods are implemented in practice, biases in, and properties of the underlying data used to train models must be accounted for (9–11). Thus, using data on its own without an understanding of who the data represents can limit validity of conclusions drawn (12). Especially given that many new data sources and technologies are opt-in and require specific technology access, these conclusions could potentially exacerbate health disparities. However, these challenges do not render the data without use. The COVID-19 pandemic has not only demonstrated potential immense utility of data for informing and evaluating large-scale public health interventions, but also the desire of many companies and data scientists to be helpful. Accordingly, we offer tangible strategies to ensure that such data are used in a manner that mitigates any added disparities that can occur by drawing inference from data without considering who is represented, but moreover how digital data and tools can be used to promote the most vulnerable. We describe three approaches for doing so, while being sensitive to privacy, legal, and organizational safeguards.

## Information That Will Illuminate Who is Represented in the Data

First, for behavior, symptom or mobility data ascertained from digital tools and used for making population-inferences, we need to better understand the denominator (the “population at risk” in epidemiology) of who is included. This denominator, including but not limited to age and gender components, would be useful to assess who is represented in any data that is shared. Understanding who is represented in the data can be useful from an evaluation perspective (e.g., so that in a pandemic situation, when evaluating the efficacy of social distancing interventions, we don't miss a small rural population that has been moving around even though the population is largely maintaining social distancing). Knowledge of the denominator also will help in identification and investigation of disparities in illness, behaviors and healthcare access within and between groups. Given that denominator information may not be available nor possible to report at an individual level due to privacy concerns, we can ensure sampling from specific populations with known attributes (13), use statistical methods to provide data weighted to represent populations equally (14), or provide denominator data at spatial resolutions that don't infringe on privacy but still illustrate differentials useful for decision making (e.g., rural vs. urban) (15). Further, it should be noted that there are important other aspects (e.g., those who report more frequently or with different thresholds than others) that may not be clear from classical demographic denominator information but can still differentiate individuals included in aggregate digital data from the average population. Toward understanding such attributes which are not easy to directly measure, tool builders could directly ask about such information (e.g., how much time do you spend online per day) or explore other features of the data that may identify these differentials. For example, social media users who talk about health-related topics may not be representative of other social media users, limiting external generalizability of conclusions. To address this limitation in studies of influenza from social media reports, researchers have contrasted participation frequency and overall topics of discussion between the two groups (those who talk about influenza symptoms and those who do not) (16).

## Utilization of Data to Understand Disparities

Second, beyond data cataloging symptoms, behaviors and mobility, other information that can be used to specifically assess the needs of vulnerable communities (e.g., the elderly) and those underrepresented in such digital symptom, behavior and mobility datasets (e.g., certain cultural or socioeconomic groups) could be shared by relevant companies and organizations. For instance, data to help us better understand who is able to access or use new tools would be important. Healthcare access, the proportion of people who typically seek care by location is an important factor in disparities. Thus, understanding how new tools such as telemedicine are being accessed by location would be useful for appropriately adjusting assessments of population illness levels from such data. Given that complete information about who is using or accessing different services may not be feasible to share due to privacy and organizational safeguards, highlighting underrepresented places or sharing data that compares data by place (e.g., relative to a high-access location) can be a first step to help us better understand which groups may be omitted from any analyses. While such data must not be used on its own to conclude for whom disparities exist at the individual level, due to the well-known ecological fallacy, such information could be used as a first step, in the absence of other information, to immediately inform public health response at the group level by identifying communities who may not be served, development of targeted surveillance mechanisms for those groups, and to understand limitations of analyses based on data in which the groups are not represented. For example, if we find that telemedicine is not being accessed by as often by people in a specific area, we can target more in-person healthcare to that region. Beyond telemedicine, data on access of other digital tools such as tool/app use or downloads by place could also be used to better understand who such data represents. Moreover, as social media has shown to be a place where people may voice opinions that are not captured through other methods such as surveys (17), or where location-specific, sub-city level, information can be captured (18), other reasons for disparities beyond access, such as specific reasons behind mistrust or preferences, by location, could be explored via passively mining data from social data feeds based on directly or indirectly related information publicly shared by individuals (19).

## Techniques to Account For Tool Use Changes in the Utilizated Data

Third, product/tool uptake must be factored in systematically when data concerning trends or patterns are shared. Technology availability and use, especially for the most vulnerable, can diverge from the overall, especially during emergency periods. This was seen, for example, during Hurricane Sandy in the northeastern United States, when the communities most impacted by the natural disaster were also the ones left without any means of communication to relay those needs (20). If information on product uptake or use cannot be directly released, changes in use should be examined in relation to outcomes and can be factored in systematically with released trends (e.g., release information on data per user, per unit time and/or location). Another way to mitigate this issue would be by identifying, or proactively recruiting, a stable subset of users which are a representative population based on the outcome of interest, on which to release trends (21).

## Summary and Conclusion

Commendably, many companies, organizations, and researchers with digital data resources have a desire to contribute to social good efforts, as we've seen in the COVID-19 acute pandemic situation. Strategies outlined here can help shed light into how these data can be put to use in close synergy with public health research and practice priorities. Not only can data be shared in a manner that refrains from advancing health disparities, but as we describe, data can be shared that could help close such gaps. Recognizing the importance of these priorities opens up new opportunities for innovation and will simultaneously strengthen the impact of digital data and tools in health research and practice.

## Author Contributions

RC conceptualized the article. All authors contributed to writing and approved the final version.

## Conflict of Interest

The authors declare that the research was conducted in the absence of any commercial or financial relationships that could be construed as a potential conflict of interest.
